# Efficacy of pirarubicin for nonmuscle invasive bladder cancer: A protocol of systematic review and meta-analysis

**DOI:** 10.1097/MD.0000000000020415

**Published:** 2020-06-05

**Authors:** Da-Yin Chen, Liang Cheng, Long-Xin Dong, Wen-Jie He, Hui-Feng Cao, Ping Wang, Cai-Fang Yue

**Affiliations:** a Department of Urology; b Department of Outpatient, The First Affiliated Hospital of Jiamusi University; c Forensic Identification Center, Criminal Technology Division of Jiamusi Public Security Bureau; d Department of Critical Care Medicine, The First Affiliated Hospital of Jiamusi University, Jiamusi, China.

**Keywords:** efficacy, nonmuscle invasive bladder cancer, pirarubicin, safety

## Abstract

**Background:**

This study will aim to appraise the efficacy and safety of pirarubicin for the treatment of patients with nonmuscle invasive bladder cancer (NMIBC).

**Methods:**

We will perform a comprehensive literature search in MEDLINE, EMBASE, Cochrane Library, Scopus, PsycINFO, Web of Science, Allied and Complementary Medicine Database, Chinese Biomedical Literature Database, and China National Knowledge Infrastructure from their beginning to the February 29, 2020. All randomized controlled trials of pirarubicin for NMIBC will be included regardless limitations related to the language and publication time. Two researchers will independently select studies from searched records, extract data from included randomized controlled trials, and assess study quality using Cochrane risk of bias tool. Any differences between them will be solved with the help of another researcher. RevMan 5.3 software will be utilized for statistical analysis.

**Results:**

This study will provide a synthesis of current evidence to investigate the efficacy and safety of pirarubicin for NMIBC using overall survival, progression-free survival, recurrence-free survival, quality of, rates of recurrence, and adverse events.

**Conclusion:**

This study will explore whether or not pirarubicin can be used as an effective and safety treatment for patients with NMIBC.

**Registration number:**

INPLASY202040113.

## 1 Introduction

Nonmuscle-invasive bladder cancer (NMIBC) is a common malignant tumor in both males and females,^[1–4]^ which accounts for about 75% of newly diagnosed bladder cancers around the world.^[5–6]^ Of those, about 10% to 20% patients are at highly risk of progression to muscle-invasive bladder cancer, and will result in very poor outcomes.^[7–8]^ Thus, effective management for this condition is very important.^[9–12]^

Over the past few years, several studies have investigated the efficacy and safety of pirarubicin for the treatment of patients with NMIBC.^[13–20]^ However, no consistent conclusions have reached among those studies. Thus, this study is designed to synthesize currently available evidences to evaluate the efficacy and safety of pirarubicin for NMIBC.

## 2 Methods and analysis

### 2.1 PROSPERO registration

We have registered this study on INPLASY202040113, and have organized this study according to the guidelines of preferred reporting items for systematic reviews and meta-analysis protocol statement.^[21]^

### 2.2 Study inclusion and exclusion criteria

#### 2.2.1 Types of studies

All randomized controlled trials (RCTs) investigating the efficacy and safety of pirarubicin for NMIBC will be included. We will exclude any other studies, except RCTs.

#### 2.2.2 Types of interventions

We will only include studies utilized pirarubicin as their interventional management.

As for a comparator, it could be any treatments, such as surgery, radiotherapy. However, studies involved pirarubicin as their control treatment is not allowed.

#### 2.2.3 Types of participants

Patients who meet the diagnosis criteria of NMIBC will be included in this study, irrespective their age, race, sex, and duration of NMIBC.

#### 2.2.4 Types of outcome measurements

Primary outcomes include overall survival and progression-free survival.

Secondary outcomes are recurrence-free survival, quality of life (as assessed by any scales reported in the trials), rates of recurrence, and adverse events.

### 2.3 Search methods for the identification of studies

A comprehensive literature search will be carried out in MEDLINE, EMBASE, Cochrane Library, Scopus, PsycINFO, Web of Science, Allied and Complementary Medicine Database, Chinese Biomedical Literature Database, and China National Knowledge Infrastructure from their initiation to the February 29, 2020. We will include any potential RCTs of pirarubicin for NMIBC regardless the language and publication time. We will create detailed search strategy for MEDLINE (Table 1), and will modify similar search strategies for other electronic databases.

**Table 1:**
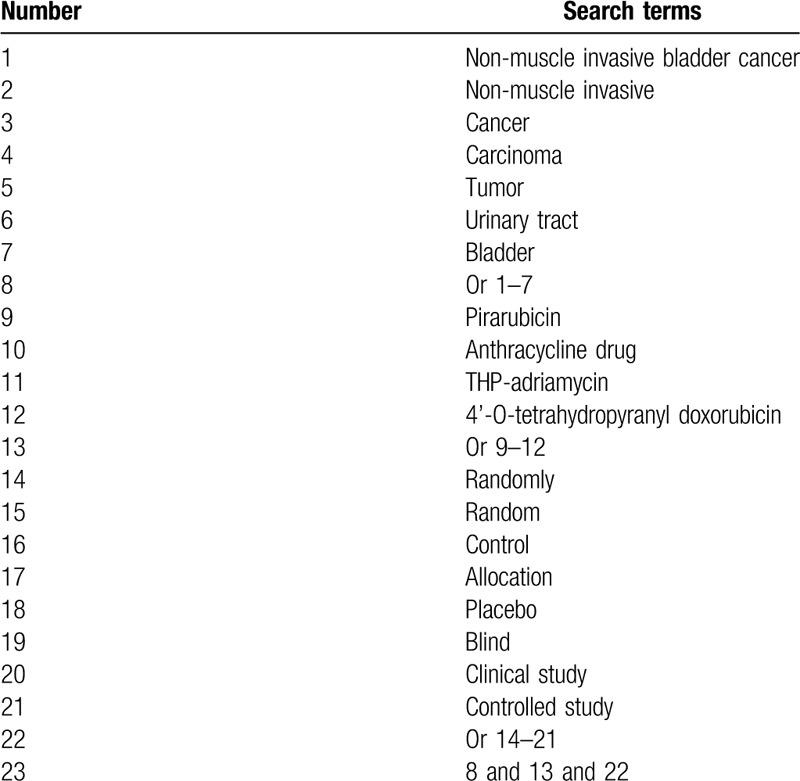
Detailed search strategy for MEDLINE.

We will also examine the websites of clinical trial registry for ongoing trials, dissertations, and reference lists of included studies.

### 2.4 Data collection and analysis

#### 2.4.1 Selection of studies

We will import all identified literatures to the EndNote X7 software and will remove all duplicated records. All titles and abstracts of potential studies will be scanned by 2 independent researchers according to the predefined eligibility criteria, and unconnected studies will be eliminated. After that, full text of the remaining studies will be examined based on all inclusion criteria. Any opposite views will be resolved by consensus with the help of another researcher. The process of study selection will be demonstrated in a preferred reporting items for systematic reviews and meta-analysis flowchart.

#### 2.4.2 Data extraction and management

Two researchers will independently perform data extraction using a previously created standardized data collection form. Any inconsistencies will be figured out with the help of another researcher, and a final decision will be reached after discussion. The extracted information comprises of publication information, author details, patient characteristics, study setting, trial design, sample size, details of intervention and controls, outcome indicators, safety data, follow-up information, results, findings, conflict of interests, and funding information. If we identify any insufficient or missing data, we will contact original authors to request them.

### 2.5 Study quality assessment

The methodological quality of all included RCTs will be appraised by 2 independent researchers using Cochrane risk of bias tool. We will assess it on 7 criteria, and will grade each one as low, unclear, or high risk of bias. Any uncertainty will be settled by another researcher through discussion.

### 2.6 Statistical analysis

We will use RevMan 5.3 software to carry out statistical analysis. We will estimate treatment effects of continuous data as mean difference or standard mean difference and 95% confidence intervals, and dichotomous data as risk ratio and 95% confidence intervals. Heterogeneity across included trials will be examined by *I*^2^ statistic: *I*^2^ ≤ 50% exerts homogeneity, while *I*^2^ > 50% suggests obvious heterogeneity. We will employ a fixed-effects model when homogeneity is found, and a meta-analysis will be conducted if it is possible. On the other hand, we will place a random-effects model if considerable heterogeneity is tested, and subgroup analysis will be performed to check the sources of such heterogeneity. If it is impossible to carry out a meta-analysis, we will undertake a narrative summary to address and report the merged outcome data instead.

### 2.7 Additional analysis

#### 2.7.1 Subgroup analysis

Subgroup analysis will be carried out to test the causes of significant heterogeneity in accordance with the differences in types of treatments, controls, and outcomes.

#### 2.7.2 Sensitivity analysis

Sensitivity analysis will be undertaken to test the robustness of merged results by omitting studies with high risk of bias.

#### 2.7.3 Reporting bias

If over 10 studies on one or more outcome indicators are included, we will attempt to find out reporting bias using funnel plot and Egger regression test.^[22–23]^

#### 2.7.4 Grading the quality of evidence

Two researchers will utilize grading of recommendation assessment, development, and evaluation method to appraise level of confidence related to the main outcomes.^[24]^ Any conflicts will be cleared up with the help of another researcher and a consensus will be made.

### 2.8 Ethics and dissemination

No ethical documents are needed in this study, because no individual patient data will be collected. We plan to publish this study on a peer-reviewed journal or presented in a conference meeting.

## 3 Discussion

Several RCTs have been conducted to investigate the efficacy and safety of pirarubicin for NMIBC. However, no study is carried out at the conceptual level. Thus, this study will evaluate the efficacy and safety of pirarubicin for NMIBC systematically. Its results may provide reference and recommendation for the clinical practice and further studies. However, this study may suffer from several potential limitations as follows: significant heterogeneity of intervention, controls and outcome measurements; low methodological quality of included studies; and small sample size of eligible trials. All those limitations may affect the findings of this study.

## Author contributions

**Conceptualization:** Da-Yin Chen, Liang Cheng, Long-Xin Dong, Hui-Feng Cao.

**Data curation:** Hui-Feng Cao, Ping Wang, Cai-Fang Yue.

**Formal analysis:** Da-Yin Chen, Liang Cheng, Wen-Jie He, Hui-Feng Cao.

**Methodology:** Da-Yin Chen, Liang Cheng, Long-Xin Dong, Wen-Jie He, Hui-Feng Cao, Ping Wang, Cai-Fang Yue.

**Resources:** Da-Yin Chen, Liang Cheng, Long-Xin Dong, Wen-Jie He, Hui-Feng Cao, Ping Wang, Cai-Fang Yue.

**Software:** Da-Yin Chen, Liang Cheng, Long-Xin Dong, Wen-Jie He, Cai-Fang Yue.

**Validation:** Da-Yin Chen, Liang Cheng, Wen-Jie He, Ping Wang, Cai-Fang Yue.

**Visualization:** Da-Yin Chen, Long-Xin Dong, Wen-Jie He, Hui-Feng Cao.

**Writing – original draft:** Da-Yin Chen, Liang Cheng, Hui-Feng Cao, Ping Wang, Cai-Fang Yue.

**Writing – review & editing:** Da-Yin Chen, Long-Xin Dong, Wen-Jie He, Hui-Feng Cao, Ping Wang.
